# Identifying and validating the roles of the cuproptosis-related gene DKC1 in cancer with a focus on esophageal carcinoma

**DOI:** 10.1007/s00432-024-05870-8

**Published:** 2024-08-05

**Authors:** Daidi Zhang, Qingwen Zhu, Xufeng Huang, Bohao Zhang, Jiaxin Zhang, Yanru Qin

**Affiliations:** 1 Department of Oncology, The First Affiliated Hospital of Zhengzhou University, Zhengzhou, 450000 Henan China; 2 Department of Otorhinolaryngology Head and Neck Surgery, The First Affliated Hospital of Zhengzhou University, Zhengzhou, 450000 China; 3 Faculty of Dentistry, University of Debrecen, Debrecen, Hungary

**Keywords:** DKC1, Diagnosis, Prognosis, Tumor microenvironment, Esophageal carcinoma

## Abstract

**Background:**

Esophageal cancer is a common malignancy of the digestive tract. Despite remarkable advancements in its treatment, the overall prognosis for patients remains poor. Cuproptosis is a form of programmed cell death that affects the malignant progression of tumors. This study aimed to examine the impact of the cuproptosis-associated gene DKC1 on the malignant progression of esophageal cancer.

**Methods:**

Clinical and RNA sequencing data of patients with esophageal cancer were extracted from The Cancer Genome Atlas (TCGA). Univariate Cox regression analysis was used to identify the differentially expressed genes related to cuproptosis that are associated with prognosis. We then validated the difference in the expression of DKC1 between tumor and normal tissues via three-dimensional multiomics difference analysis. Subsequently, we investigated the association between DKC1 expression and the tumor microenvironment by employing the TIMER2.0 algorithm, which was further validated in 96 single-cell datasets obtained from the TISCH database. Additionally, the functional role of DKC1 in pancarcinoma was assessed through GSEA. Furthermore, a comprehensive pancancer survival map was constructed, and the expression of DKC1 was verified in various molecular subtypes. By utilizing the CellMiner, GDSC, and CTRP databases, we successfully established a connection between DKC1 and drug sensitivity. Finally, the involvement of DKC1 in the progression of esophageal cancer was investigated through in vivo and in vitro experiments.

**Results:**

In this study, we identified a copper death-related gene, DKC1, in esophageal cancer. Furthermore, we observed varying levels of DKC1 expression across different tumor types. Additionally, we conducted an analysis to determine the correlation between DKC1 expression and clinical features, revealing its association with common cell cycle pathways and multiple metabolic pathways. Notably, high DKC1 expression was found to indicate poor prognosis in patients with various tumors and to influence drug sensitivity. Moreover, our investigation revealed significant associations between DKC1 expression and the expression of molecules involved in immune regulation and infiltration of lymphocyte subtypes. Ultimately, the increased expression of DKC1 in esophageal cancer tissues was verified using clinical tissue samples. Furthermore, DKC1-mediated promotion of esophageal cancer cell proliferation and migration was confirmed through both in vitro and in vivo experiments. Additionally, it is plausible that DKC1 may play a role in the regulation of cuproptosis.

**Conclusion:**

In this study, we conducted a systematic analysis of DKC1 and its regulatory factors and experimentally validated its excellent diagnostic and prognostic abilities in various cancers. Further research indicated that DKC1 may reshape the tumor microenvironment (TME), highlighting the potential of DKC1-based cancer treatment and its usefulness in predicting the response to chemotherapy.

**Supplementary Information:**

The online version contains supplementary material available at 10.1007/s00432-024-05870-8.

## Introduction

Esophageal carcinoma (ESCA) is a common tumor of the digestive tract (Sung et al. 2021). Although remarkable progress has been made in its treatment, the overall prognosis for patients remains poor (Shah et al. 2023). Once it relapses or metastasizes, the 5-year survival rate decreases to less than 20% (Kurogochi et al. 2022). Therefore, studying the molecular mechanism underlying the malignant progression of esophageal cancer is crucial for guiding its treatment and prognosis.

Copper (Cu) is an indispensable trace element in the body that plays important roles in a myriad of biological processes, including cell proliferation, angiogenesis, and metastasis of malignant tumors (Cui et al. 2021; Davis et al. 2020; O'Day et al. 2009; Zheng et al. 2022).

The DKC1 gene is involved in the synthesis of telomerase and reverse transcriptase. Recent studies have revealed that it is aberrantly expressed in a wide range of cancers and is involved in cancer development and progression (Elsharawy et al. 2020; Hou et al. 2020). By systematically investigating the aberrant expression of the DKC1 gene in cancers, it is possible to elucidate the mechanisms underlying the role of this gene in cancer. Currently, the understanding of the involvement of DKC1 in ESCA, its prognostic significance, and its association with tumors is limited. Therefore, the objectives of the present study were to identify DKC1 associated with ESCA and to investigate the relationship between DKC1 and clinical outcome, clinical features, the TME, and antitumor therapy. Moreover, we aimed to determine the involvement of DKC1 in ESCA by conducting in vivo and in vitro experiments, revealing a novel perspective for the clinical treatment and diagnosis of ESCA.

## Methods

### Datasets and source

This study utilized diverse datasets obtained from publicly available databases to investigate various aspects of cancer. The TCGA pancancer cohort provided valuable resources, including mRNA expression, copy number variation, masked copy number partitioning, and DNA methylation 450 K data. These datasets were accessed through the Firehose database, which offers unrestricted access to both tumor and normal samples (http://gdac.broadinstitute.org). Additionally, the UCSC Xena database was utilized to acquire miRNA, TCPA, mutation data, molecular subtyping, and clinical data (https://xenabrowser.net/datapages/). The GEO database was used for validating external mRNA-level data (https://www.ncbi.nlm.nih.gov/geo/), while the CPTAC database contains mass spectrometry data at the protein level (https://proteomics.cancer.gov/programs/cptac). Immunohistochemistry and immunofluorescence data were obtained from the HPA database. For the evaluation of the pancancer immune infiltration results, the TIMER 2.0 database provided multiple immune infiltration algorithms (http://timer.cistrome.org/).

It is important to highlight that these aforementioned public databases are freely accessible, and the study strictly adhered to the policies set by each database during the data extraction process. Consequently, no ethical review or approval was necessary.

### Multiomics differential expression analysis

The primary objective of this investigation was to explore the dysregulation of DKC1 gene expression in tumors and normal tissues and to derive correlation results via multidimensional difference analysis. Due to the insufficient number of normal tissue samples in the TCGA dataset, we integrated data from the GTEx to increase confidence and sample size. We used the Wilcoxon test for difference detection, and the significance level was set at p < 0.05. To better understand the distribution of DKC1 gene expression in different organs, we used the “gganatogram” package for visualization analysis. Next, in this study, a comparison was made to assess the differences in DKC1 mRNA expression between tumor tissues and adjacent normal tissues in TCGA, and a Wilcoxon test analysis was performed on paired samples in the TCGA cancer subgroup. To assess the importance of the DKC1 gene in a wide range of cancer diagnostics, we used the “pROC” package to calculate the area under the curve (AUC) values.

### External validation and immunohistochemistry in clinical tissues

In this study, we used multiple validation methods. We used the GEO database to validate the accuracy of the gene expression data. To ensure the validity and accuracy of the findings, we also used CPATAC-based mass spectrometry data and immunohistochemistry data from the HPA database to validate protein expression.

### SCNA, mutation and DNA methylation analysis

cBioPortal is a comprehensive database for cancer genomics-related data that provides various querying and visualization tools for multiple types of genomic data, including somatic mutation, DNA copy number alteration (CNA), and DNA methylation data (Cerami et al. 2012). In the analysis of copy number alterations (CNAs) and mutations, we employed heterozygosity and homozygosity states to determine the copy number variation of each gene, defining genes with alterations in more than 5% of the samples as high-frequency CNAs. Spearman correlation was utilized to assess the relationship between CNAs and the expression of the DKC1 gene, and the degree of association between gene expression values and copy number segment values was calculated.

Furthermore, we used the R package “IlluminaHumanMmethylation-450kanno.ilmn12. hg19” from Bioconductor to annotate the methylation status of each gene’s promoter. Through the Wilcoxon rank sum test, we performed differential methylation testing on each gene in tumor samples and normal samples to identify genes exhibiting significantly lower or higher methylation. Finally, we computed the Spearman correlation between gene transcription and promoter DNA methylation beta values. To identify significant correlations, a p value threshold of less than 0.05 was adopted as the statistical criterion in this study. We utilize cBioPortal to select the “TCGA Pan Cancer Atlas Study”^12^ and input the DKC1 gene for a feature search to evaluate the association between copy number alterations (CNAs) and mutations in the DKC1 gene and its expression.

### TME and validation through single-cell analysis

The TME exerts a fundamental influence on the progression and pathogenesis of malignant tumors, with interactions between tumor cells being of utmost importance. To gain deeper insights into these interactions, a comprehensive investigation was undertaken to examine the potential associations between the expression of DKC1 and genes related to immune responses. In this study, we calculated the TIP score and subsequently conducted a comparative analysis of the differences in TIP between two distinct groups categorized based on the expression levels of the DKC1 gene. To assess the significance of the observed differences, we employed the Wilcoxon test. For a more accurate assessment of immune infiltration levels, we utilized the TIMER2.0 platform and analyzed the TCGA tumor spectrum using seven advanced algorithms, namely, TIMER, XCELL, MCPCOUNTER, QUANTISEQ, EPIC, CIBERSORT, and CIBERSORT_ABS. Furthermore, we validated the results of the TME by leveraging single-cell datasets from the TISCH database and obtained gene expression profiles across different single-cell datasets. In summary, we performed a comprehensive analysis and visualization of a pancancer cohort to explore the relationship between the TME and gene immune infiltration.

### Pathway and functional mechanism analysis

To investigate the relationships between genes and different types of tumors, a series of analyses were conducted. First, based on the DKC1 expression levels, the tumor samples were stratified into two distinct groups. Then, GSEA was employed to explore the activity of gene sets within these groups, including the activation or inhibition status of 50 hallmark gene sets and 74 metabolism gene sets. Additionally, single-cell analysis data were processed using the CancerSEA (Yuan et al. 2019) website, and 14 functional states were redefined. The z score algorithm was utilized to calculate the gene set activity for these functional states, reflecting the pathway activity of interest, and the correlation between genes and functional states was evaluated through pearson correlation analysis. Furthermore, the relationships between the six cell death scores and gene mRNA expression levels were investigated. To gain insight into the potential impact of DKC1, a comprehensive analysis utilizing differential KEGG enrichment was conducted to elucidate the specific pathways that they may modulate. Protein‒protein interaction data were screened, and proteins interacting with DKC1 were determined using protein localization and interaction scores (Veres et al. 2015). Finally, gene-related proteins were identified using CRISPR knockout screen-derived gene effect scores, and the associations between genes and proteins were systematically studied using Spearman correlation analysis.

### Analysis of clinical variables and molecular subtypes

To explore the differences in the gene expression levels of DKC1 across diverse tumor subtypes, we used the Kruskal‒Wallis test. Additionally, we used the chi-square test as a statistical method to compare and evaluate differences in molecular subtypes and clinical characteristics between groups with high and low DKC1 expression. Furthermore, we accessed comprehensive cancer immunosubtype information from the USCS Xena database, encompassing wound healing type (C1), IFN-γ-dominant type (C2), inflammatory type (C3), lymphocyte-depleted type (C4), immune quiescent type (C5), and TGF-β-dominant type (C6). These subtypes provided detailed descriptions of the tumor’s immune status. We assessed the variations in DKC1 expression across diverse molecular subtypes of tumors and subsequently investigated the associations between groups exhibiting divergent levels of DKC1 expression in relation to molecular subtypes and clinical characteristics.

### Survival and clinical outcome analysis

This study investigated the relationships between DKC1 gene expression and several prognostic indicators, including disease-specific survival (DSS), overall survival (OS), the progression-free interval (PFI), and the disease-free interval (DFI). And we searched the survival data using the TCGA database, applied the “survival” and “survminer” packages in R, and used two statistical methods, namely, the Kaplan‒Meier method and one-way Cox analysis, to comprehensively evaluate the relationship between DKC1 and tumors. In the Kaplan‒Meier survival analysis, we determined the optimal cutoff values for the different DKC1 mRNA expression groups using the “survminer” package and performed a log-rank test using the Surfeit function to assess significant differences. In addition, we used the “forestplot” package to visualize the results of the Cox analysis.

### Identification of chemical compounds that interact with DKC1

The Comparative Toxicogenomics Database (CTD) (Davis et al. 2021) provides toxicological information on chemical substances, genes, phenotypes, diseases, and exposures, which helps us gain a deeper understanding of their potential impacts on health. Additionally, we performed gene expression and drug sensitivity analyses using the GSCALite database (Liu et al. 2023). This database includes information on 750 small-molecule drugs and combines it with gene expression data to reveal valuable insights into the associations between these drugs and DKC1. To further explore the relationship between drugs and DKC1 expression, we used a cancer cell line platform created by the National Cancer Institute (NCI) of the United States, which includes 60 human cancer cell lines covering nine different types of cancer. By analyzing the DKC1 expression data and drug sensitivity scores of these cell lines, we calculated Spearman correlation coefficients and identified genes whose expression significantly differed among various cancer types. From these findings, we selected 150 cancer-related genes as markers and conducted matching score calculations using the CMAP_gene_signatures. RData file (Malta et al. 2018; Yang et al. 2022). Finally, we summarized and visualized the research results for 32 cancer types using the R programming language.

### Colony formation assay

For the colony formation assay, EC9706 and KYSE30 cells were transfected with negative control and ShDKC1 (2 × 10^2^ cells/well) in a 6-well plate (Corning, NY, USA) and cultured for two weeks. Afterward, the colonies were stained with 0.01% crystal violet for 10 min.

### Transwell migration assay

For the Transwell migration assay, EC9706 and KYSE30 cells were plated into the upper chambers of Transwell units (pore size, 8 μm, Corning). The lower chamber was filled with DMEM medium supplemented with 20% FBS. The Transwell units were incubated for 36 h and stained for 5 min. Cell migration onto the lower surface was quantified through microscopic examination.

### Cell counting Kit-8 (CCK8) assay

Logarithmic growth phase ESCA cells EC9706 and KYSE30 were seeded into a 96-well plate. After cell adhesion, the culture medium containing different concentrations (0, 20, 100, 200, 400, 800 μM) of Fasudil was added to the esophageal cancer cells and incubated for 48 h. Then, the medium containing the drug was carefully aspirated along the walls of the 96-well plate, and 100 μL of freshly prepared medium containing 10% CCK-8 was added to each well. The plate was placed in a cell culture incubator at 37 °C for 2 h while avoiding light exposure. The absorbance (OD) value at 450 nm was measured using a microplate reader, and the half-maximal inhibitory concentration (IC50) of Fasudil was calculated.

### RNA collection and qPCR

The clinical samples in this study of biological tissue originated from the First Affiliated Hospital of Zhengzhou University. This study has been approved by the Ethics Committee of Zhengzhou University. Tissue RNA Purification Kit Plus (EScience Biotech, Shanghai, China) was used to obtain RNA samples from nine ESCA tissue cases and eight adjacent tissue cases as the manufacturer’s instructions. Subsequently, the RNA was converted into cDNA using the UEIris qPCR System for First-Strand cDNA Synthesis. For qPCR amplification, the Applied Biosystems QuantStudio5 (Thermo Fisher Scientific, MA, USA) was used in a 20 μL reaction volume, which included 10 μL of Universal SYBR Green qPCR Supermix (US Everbright, Suzhou, China). The primers were synthesized by Sangon Biotech (Shanghai, China), and PCR was repeated three times for validation. The mRNA expression levels were normalized to GAPDH expression values. The utilized primer pairs were as follows: GAPDH: 5′-ATCACCATCTTCCAGGAGCGAG-3′ (forward), 5′-CATTGCTGATGATCTTGAGGCTGT-3′ (reverse); DKC1: 5′-TAACATGGCGGATGCGGAAG-3′ (forward), 5′-TCAGCGTGTTGTATTTCGGCTTGT-3′ (reverse).

### Immunohistochemistry (IHC)

DKC1 antibody from Proteintech (Cat. 25420–1-AP, Wuhan, China) was used for immunohistochemistry on 27 ESCA tissue samples and 29 corresponding adjacent normal tissue samples obtained from the First Affiliated Hospital of Zhengzhou University. SPlink Detection Kits (Origene, Beijing, China) were used for IHC of DKC1(diluted at 1:500). An EDTA-based pH 9.0 solution was used for retrieval. The images were captured using a Zeiss Axio Scope 5 microscope.

### Tumor xenograft model

BABL/c male nude mice aged 4–6 weeks were obtained from Gempharmatech Company (China) and housed in isolator cages under specific pathogen-free (SPF) conditions. The animal procedures adhered to the guidelines endorsed by the Institutional Animal Care and Use Committee of Zhengzhou University, ensuring ethical conduct and compliance (Approval NO. ZZU-LAC2020111707). Mice were provided with a sterilized chow diet and water ad libitum. A total of 1.5 × 10^6^ stably transfected vector control or EC9706 ShDKC1 cells were subcutaneously injected into the mice. Tumor volumes were measured twice a week.

### Statistical analysis

Statistical analysis was conducted using GraphPad Prism 7 software with Student's t test and one-way ANOVA.

## Results

### Identification of cuproptosis-related mRNAs (crmRNAs) with prognostic value in ESCA

**Fig. 1:**
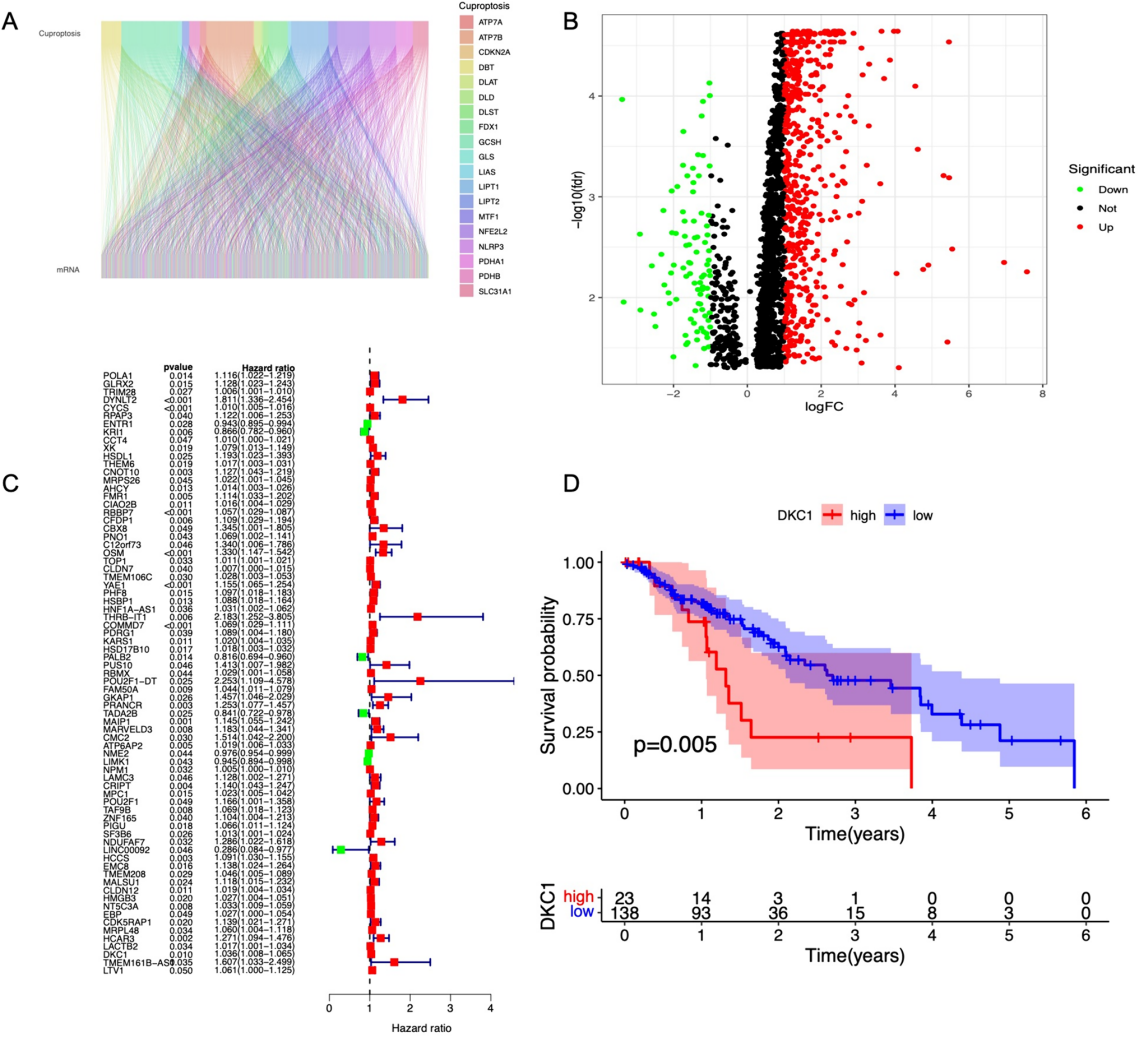
Identification of differentially expressed cuproptosis-related mRNAs (crmRNAs) with prognostic value in ESCA. **A** Sankey plot displaying the network of coexpressed CRGs and crmRNAs (R^2^ > 0.4 and *P* < 0.05). **B** Volcano plot showing the differential crmRNAs among normal and tumor samples, with red representing upregulated crmRNAs. **C** Forest plot showing the prognostic crmRNAs in ESCA. **D** Relationship between DKC1 expression and prognosis in ESCA patients

We first obtained the expression profiles of 19 copper death-related genes and esophageal cancer mRNAs from the TCGA database. Pearson correlation analysis revealed 4271 copper death-related genes (R^2^ > 0.4, p < 0.05) (Fig. 1A). Subsequently, 1448 DEGs were identified through differential gene expression analysis between normal and tumor samples (Fig. 1B). We then identified 75 prognostic genes through univariate Cox analysis (Fig. 1C). Among these prognostic genes, we focused on DKC1, a conserved gene that encodes the RNA-binding protein dyskerin and is an important component of the telomerase holoenzyme. We validated the prognostic value of DKC1 in esophageal cancer patients and found that patients with high DKC1 expression had a worse prognosis than those with low DKC1 expression (Fig. 1D).

### Pancancer expression of DKC1

**Fig. 2:**
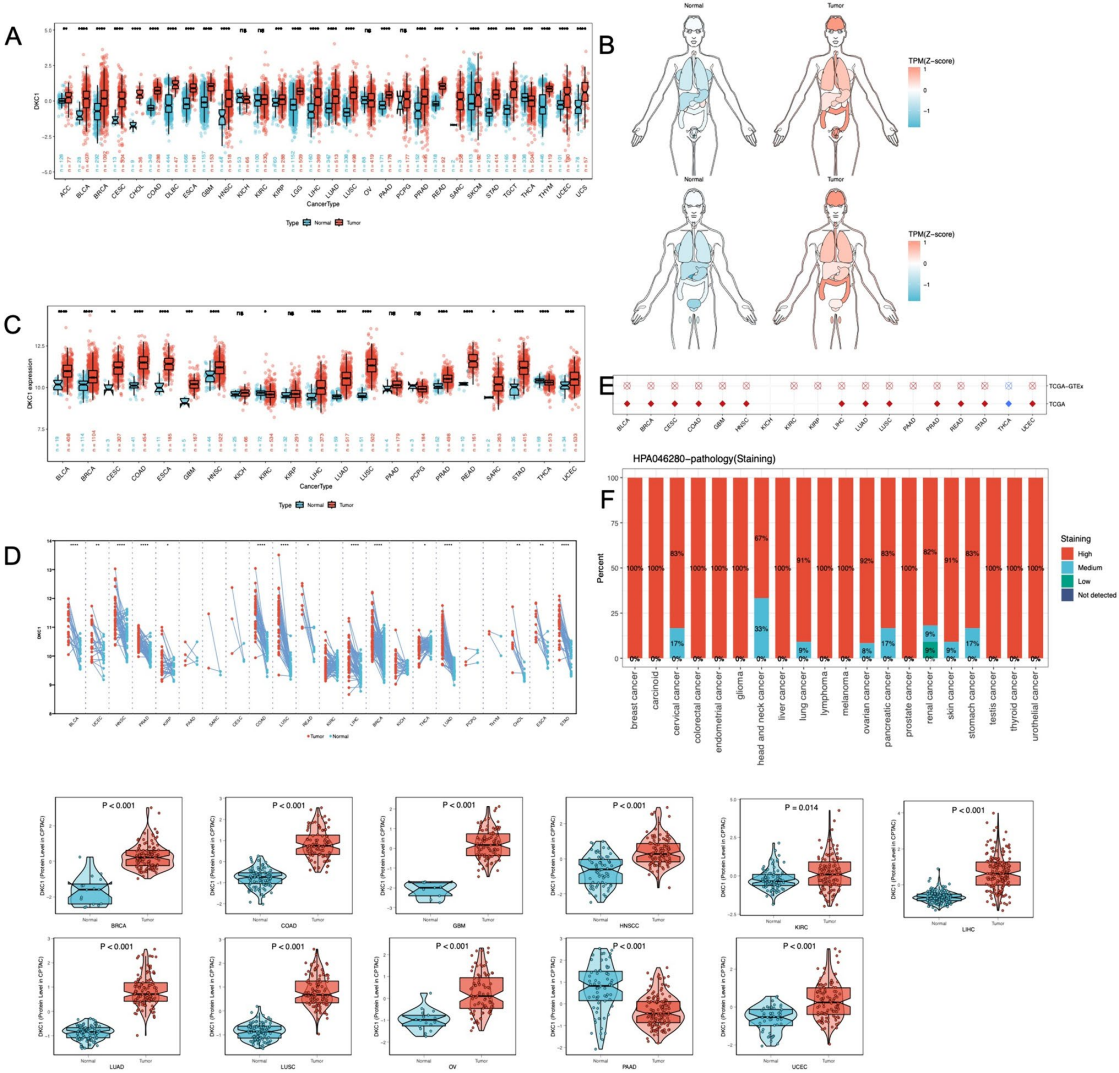
The expression landscape of DKC1. **A** The expression levels of DKC1 in various tumor tissues and their corresponding normal tissues. **B** Expression and distribution of DKC1s in various organs. **C** Y-axis representing DKC1 mRNA expression in the TCGA cohort. Boxplots show the median, quartiles, min, and max, with each point representing one sample. p values are based on the Wilcoxon test. **D** Similar to **C** but in paired samples grouped by cancer from the TCGA. Each point represents one sample. (*P < 0.05, **P < 0.01, ***P < 0.001, ****P < 0.0001). **E** Logistic regression analysis of both TCGA and TCGA-GTEx data. Red indicates that the OR is greater than 1, and blue indicates that the OR is between 0 and 1. **F** Staining of DKC1 in tumor tissues based on the HPA. **G** Analysis of the protein level of DKC1 in the CPTAC mass spectrometry database

To determine the pattern of DKC1 dysregulation in cancer, we expanded the normal sample size by combining and mining resources from the TCGA and GTEx databases. We comprehensively investigated DKC1 expression levels in cancer (Fig. 2A, Supplementary Table 1). The expression distribution pattern was visualized using organ graphs (Fig. 2B). We found that DKC1 was significantly upregulated in several cancers, such as lung adenocarcinoma (LUAD), liver hepatocellular carcinoma (LIHC), and breast invasive carcinoma (BRCA), and exhibited widespread upregulation across different cancer types. Subsequent differential analysis of TCGA samples (Fig. 2C) and paired differential analysis (Fig. 2D), along with logistic regression analysis (Fig. 2E), provided strong validation of the above results. Moreover, the estimated ROC curve (Supplementary Fig. 1) indicated that DKC1 mRNA expression had satisfactory sensitivity and specificity for diagnosing 16 types of cancer (AUC > 0.7).

**Fig. 3:**
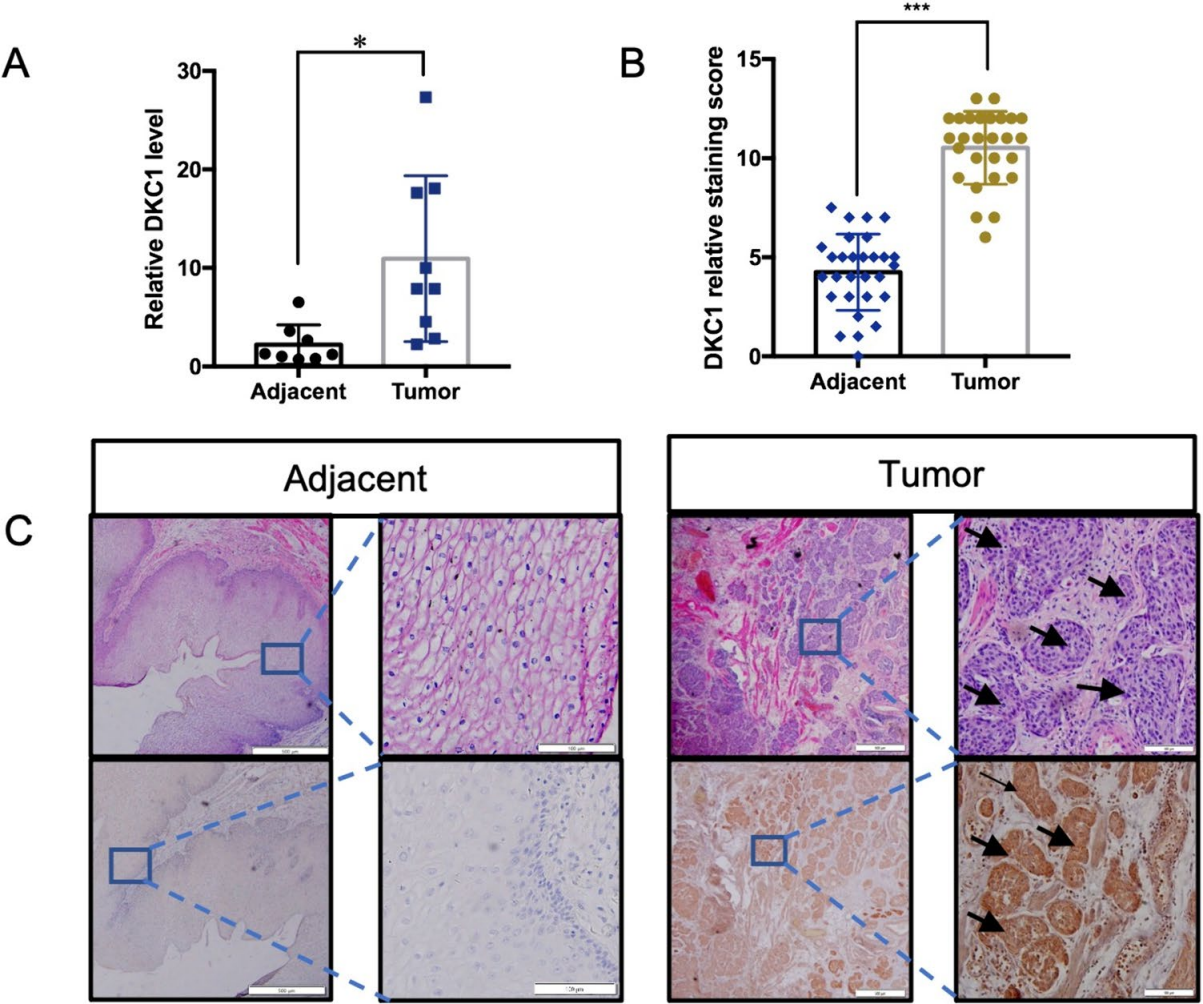
DKC1 is highly expressed in ESCA. **A** qPCR detection of DKC1 transcript expression levels in ESCA tumor (n = 9) and adjacent normal (n = 8) tissues. **B** Protein expression levels were compared between ESCA tumor(n = 27) and adjacent normal (n = 29) tissues. **C** Representative images of DKC1 IHC staining in ESCA tumor and adjacent normal tissues. All data were analyzed with the unpaired t-test. **P < 0.01, ***P < 0.001. Error bars represent the mean ± SD

After expanding the normal sample size by combining the GTEx database, the results remained robust (Supplementary Fig. 2). DKC1 mRNA expression was further validated using the GEO database (Supplementary Fig. 3). Proteomic analysis indicated a direct correspondence between the expression of the DKC1 protein and its transcriptional regulation (Fig. 2G). We presented the statistical results of immunohistochemical analysis of DKC1 expression in the HPA database. Overall, DKC1 exhibited high expression in all tumor tissues within the database (Fig. 2F). To validate the presence of DKC1 in ESCC clinical tissues, we conducted qPCR and IHC investigations. The findings indicated that DKC1 was markedly increased in ESCC tissues when compared to adjacent non-tumour samples (Fig. 3).

### Genetic alterations of DKC1 in cancers

**Fig. 4:**
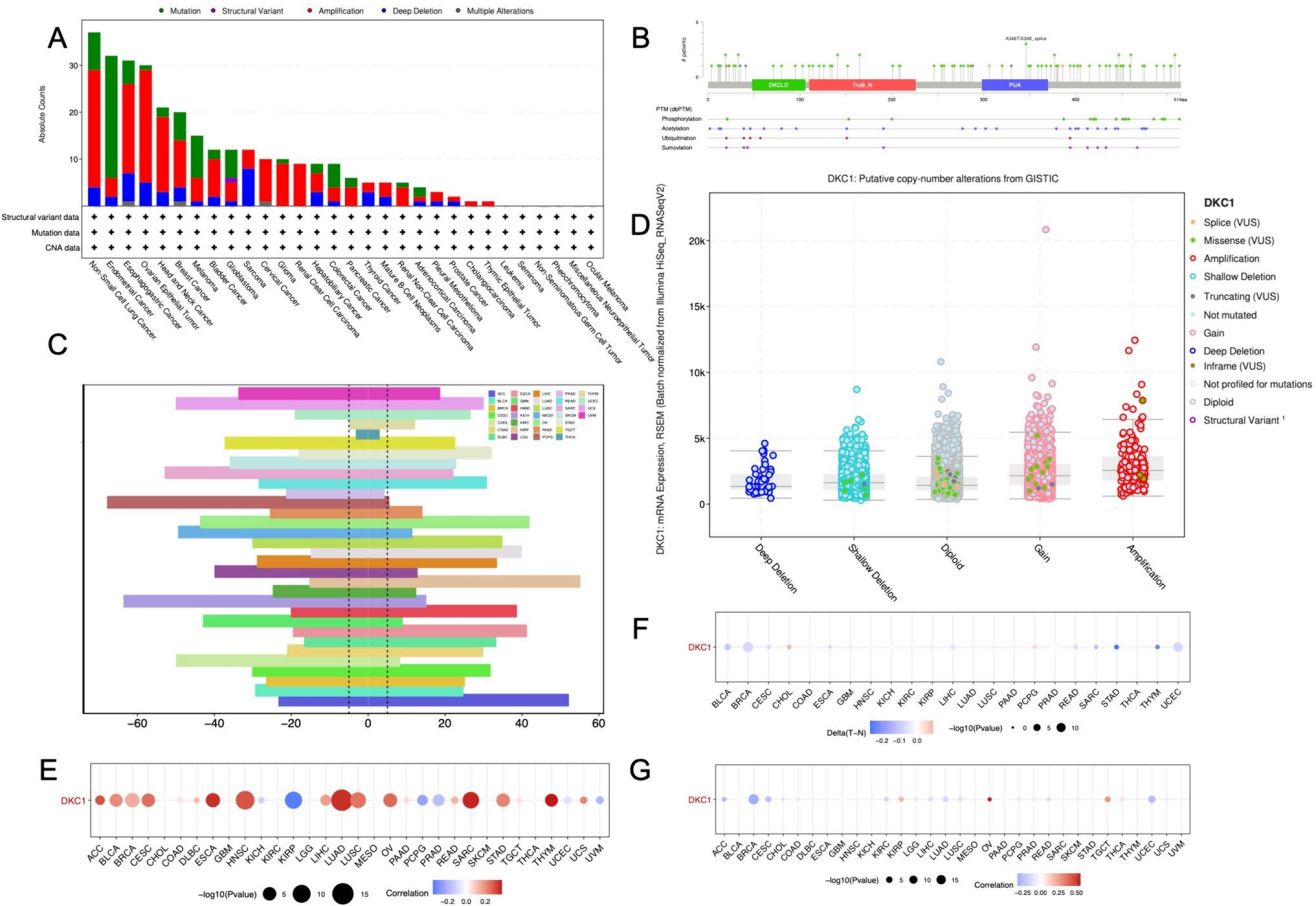
Genetic alterations of DKC1 in cancers. **A** Pancancer sites and numbers of patients with DKC1 genetic alterations according to cBioPortal. **B** The structure of DKC1 mutation sites. **C** Frequency of DKC1 mutations in different tumor types. **D** Relationship between DKC1 mRNA expression and genetic alterations. **E** Spearman’s correlation between somatic copy number alterations and DKC1 expression. **F** Heatmap showing the differential methylation of DKC1 in cancers; hypermethylated and hypomethylated DKC1 are marked in red and blue, respectively (Wilcoxon rank-sum test). **G** Spearman’s correlation of DKC1 transcription and promoter methylation. Red and blue represent positive and negative correlations, respectively

We analyzed genomic data obtained from both tumors and normal tissue samples collected from the TCGA pancancer cohort, including genetic variations, somatic copy number alterations (SCNA), mRNA expression, and DNA methylation data. We systematically examined gene mutation sites (Fig. 4B). According to the cBioPortal database, genetic alterations were found to be relatively infrequent across various cancer types. Amplification predominated in multiple tumors, including ESCA (Fig. 4A). SCNA plays a critical role in regulating DKC1 expression in tumors (Fig. 4E). To delve deeper into genetic abnormalities in cancer, we analyzed the proportion of SCNAs. Generally, SCNA occurred with a high frequency in most cancer types (in over 5% of all samples), with only a few tumors showing a low frequency (Fig. 4C). Thus, we investigated the effect of SCNA on DKC1 mRNA expression by analyzing the Spearman correlation between DKC1 expression and the masked copy number segment of TCGA. In the majority of tumors, a significant correlation was observed between DKC1 mRNA expression and SCNA. This result suggests that gene copy number aberrations are common in most cancers and can affect DKC1 expression. We observed complex methylation patterns of the gene in the pancancer cohort (Fig. 4F, G). Although the methylation pattern of DKC1 varied, an overall negative correlation was observed between DKC1 mRNA expression and DNA methylation, suggesting the complexity and cancer specificity of DKC1 regulation.

### Associations between DKC1 and pathways in cancer

**Fig. 5:**
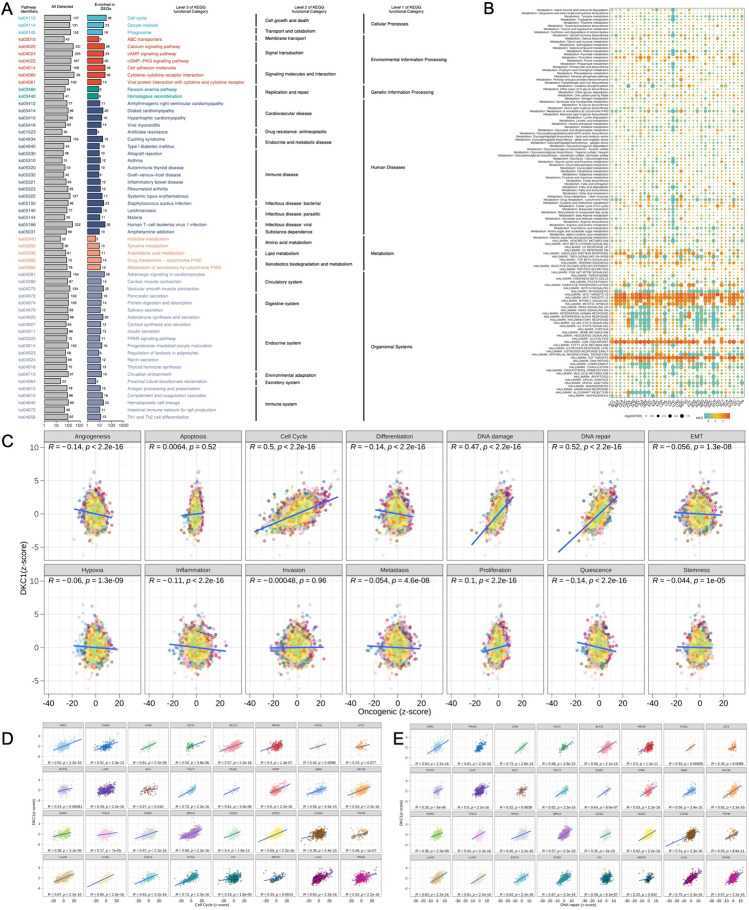
Pathway and functional mechanism analysis. **A** The enrichment results of KEGG analysis. **B** DKC1 mRNA expression was strongly correlated with 14 malignant features of all tumors. Cell cycle and DNA repair scores were generally positively correlated with DKC1 mRNA expression. **C** Differences in the enrichment of DKC1 in 50 HALLMARK and 74 metabolic gene sets. NES is the normalized enrichment score in the GSEA algorithm. **D**, **E** Correlation scatter plot of the cell cycle distribution and DNA repair

We analyzed the correlation between hallmark scores and genes in 14 cancers, where DNA repair and cell cycle-related scores showed a higher positive correlation coefficient than other scores (Fig. 5B), consistent with pathway analysis (Fig. 5A). In addition, we systematically analyzed metabolic pathways and found good consistency among different cancers, suggesting the conservation of gene function. Tumors exhibiting elevated DKC1 expression commonly displayed enrichment in cell cycle-related pathways (Fig. 5A, C), as supported by the HPA database, which indicated that the gene had cell cycle-dependent expression and transcriptional changes related to the cell cycle (Fig. 5D, E). We also systematically analyzed proteins associated with gene mRNA expression (Supplementary Table 2). The comPPI helped us identify genes that may interact with DKC1 (Supplementary Fig. 4, Supplementary Table 3).

### Correlation between DKC1 expression and immune infiltration

**Fig. 6:**
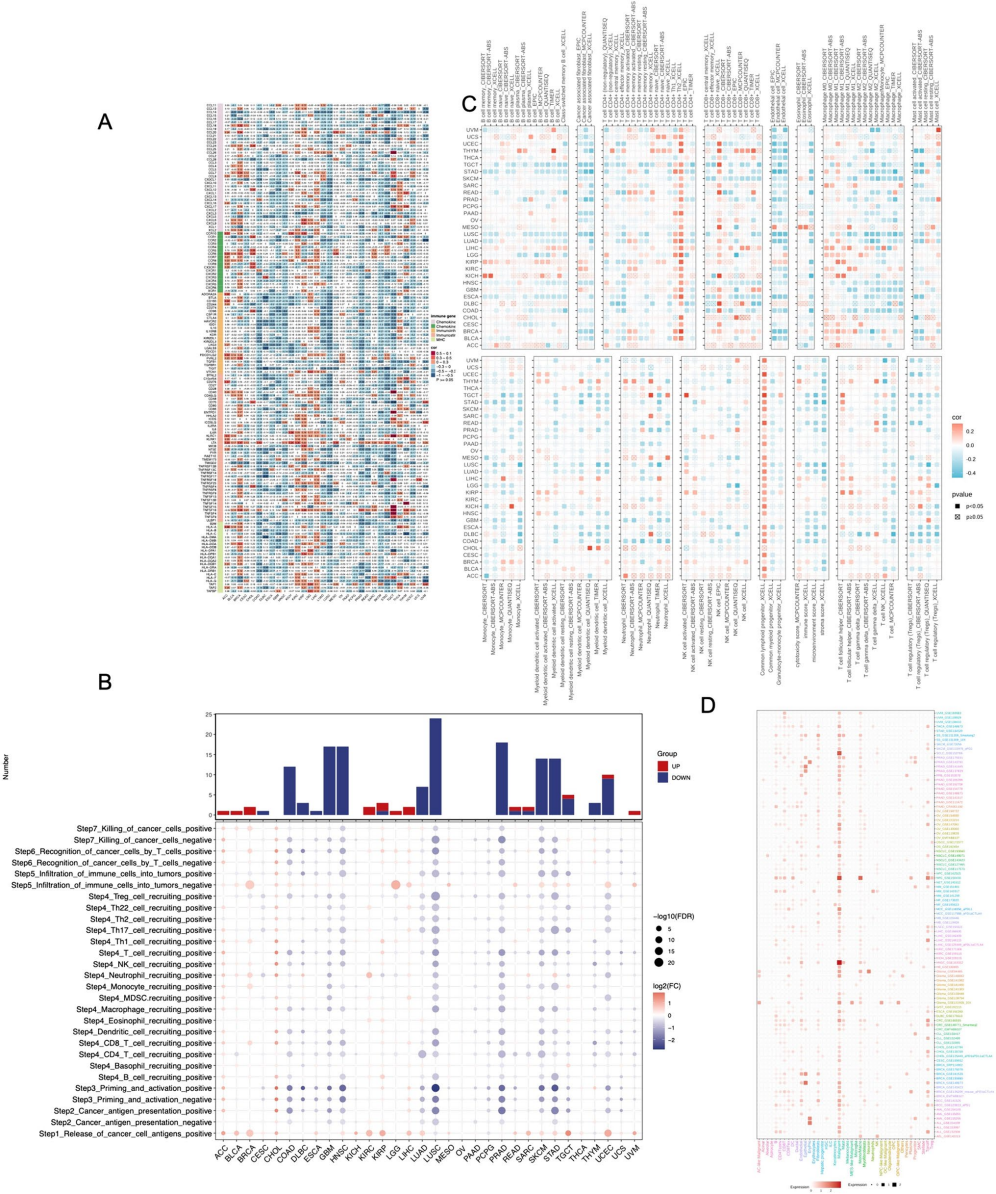
Association of DKC1 expression with immune infiltration. **A** Heatmap showing correlations between DKC1 mRNA expression and the expression of chemokines, chemokine receptors, immune inhibitors, immune stimulants, and major histocompatibility complex (MHC) genes. **B** The bubble map shows the difference in the TIP score. The size represents significance, the depth of color represents logfc, blue represents a decrease, and red represents an increase. **C** Seven software programs were used to evaluate the correlation between DKC1 expression and cancer immune infiltration. **D** Pancancer cell sources of DKC1 at the single-cell level. The heatmap shows the distributions of DKC1 expression in different cells among 24 types of cancer

To investigate whether DKC1 is involved in the immune infiltration process in cancer, we assessed the associations between DKC1 and major histocompatibility complex (MHC) molecules, immune checkpoint genes, immune activation genes, and chemokines (Fig. 6A). The results revealed complex cancer-specific patterns, with significant positive correlations observed in some tumors, such as adrenocortical carcinoma (ACC), BRCA, kidney renal clear cell carcinoma (KIRC), kidney renal papillary cell carcinoma (KIRP), LIHC, testicular Germ Cell Tumors (TGCT), Thyroid carcinoma (THCA), while significant negative correlations were observed in other tumors, such as colon adenocarcinoma (COAD), lymphoid Neoplasm Diffuse Large B-cell Lymphoma (DLBC), ESCA, glioblastoma multiforme (GBM), lung squamous cell carcinoma (LUSC). Th1, Th2, M0, and lymphoid progenitor cells were positively correlated with DKC1 mRNA expression, whereas CD4 + T central memory, effector memory, and memory resting cells, CD8 + T central memory and effector memory cells, and NK cells were negatively associated with DKC1 mRNA expression. These results suggest that DKC1 is involved in immune infiltration suppression and the formation of an immune desert-type TME and plays a critical role in immune-tumor interactions. However, the consistency of the analysis was confirmed by seven software tools which ensured the accuracy of our study (Fig. 6C). The TIP score reflects the immune activity of tumors, and in most tumors, DKC1 mRNA expression is negatively correlated with immune activity (Fig. 6B). Furthermore, the TISCH database describes a consistent expression landscape of the gene in 72 single-cell datasets, including 31 cancer types, indicating that DKC1 is mainly expressed in malignant cells, T cells, and monocytes (Fig. 6D).

### Associations of DKC1 with molecular subtypes and clinical stages

**Fig. 7:**
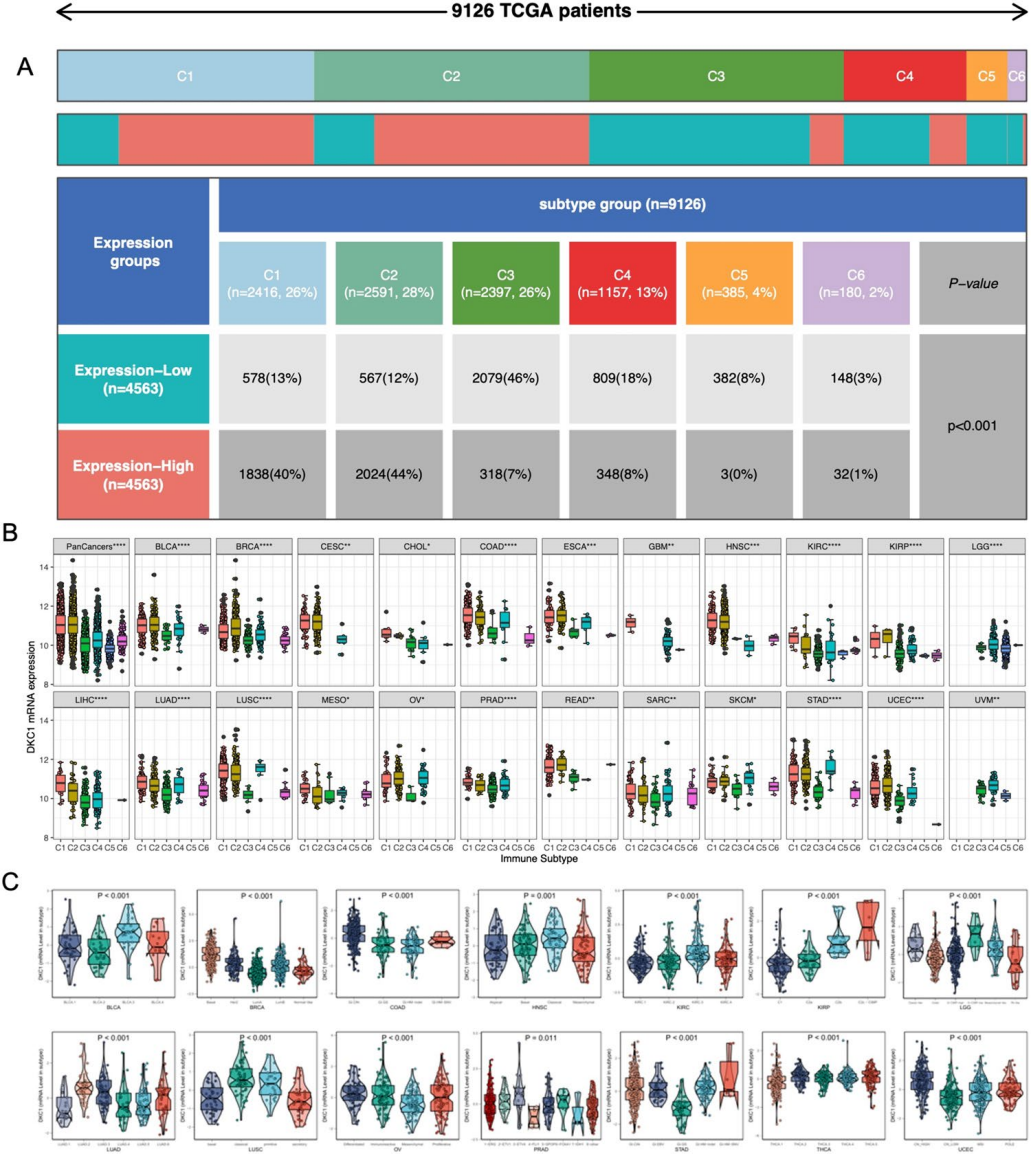
Analysis of clinical variables and molecular subtypes. **A** The chi-square test confirmed that there were more patients with the C1 and C2 subtypes in the DKC1 high-expression group and more patients with the C3 subtype in the DKC1 low-expression group. **B** Expression levels of DKC1 in different immune subtypes. The Kruskal test detected differences between the 6 immune subtypes. **C** The Kruskal‒Wallis test was used to examine differences in DKC1 expression among molecular subtypes

The expression of the DKC1 gene exhibited a positive correlation with the stage of 5 tumors (Supplementary Fig. 5), suggesting a potential association between the DKC1 gene and tumor progression. Subsequent chi-square test analysis revealed a greater number of patients in the high DKC1 expression group (C1 and C2 subtypes), while a greater proportion of patients in the low DKC1 expression group were in the C3 subtype (Fig. 7A). Furthermore, the Kruskal‒Wallis test results indicated relatively greater expression of the DKC1 gene in the C1 and C2 subtypes, whereas its expression was lower in the C3 subtype (Fig. 7B). Additionally, in multiple types of tumors, the expression levels of the DKC1 gene significantly differ among different molecular subtypes (Fig. 7C). These findings provide important insight for further exploration of the relationship between the DKC1 gene and tumor development.

### Clinical relevance of DKC1

**Fig. 8:**
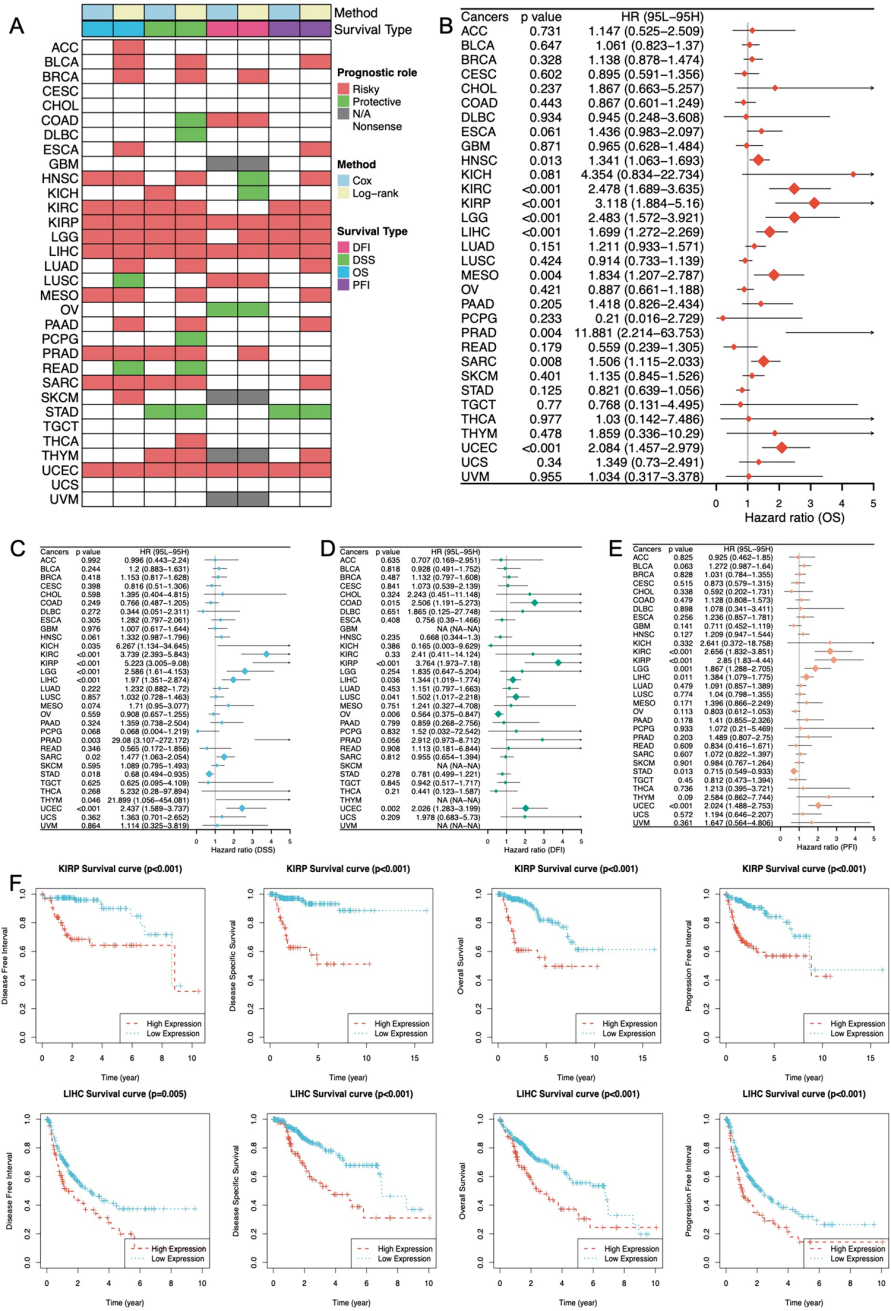
Pancancer survival landscape. **A** Association between DKC1 expression and various clinical outcomes, including OS, DSS, DFI, and PFI. Red indicates a higher risk, while green represents a protective factor. **B**–**E** The forest plot shows the prognostic significance of DKC1 in various cancer types according to univariate Cox regression analysis. **F** Kaplan‒Meier survival analysis and log-rank tests were performed using the “survival” and “survminer” packages

To further investigate the clinical significance of DKC1 in cancer, we analyzed its role in cancer survival. By analyzing a pancancer survival map, this study demonstrated the relationship between DKC1 expression and survival in patients with various cancer types (Fig. 8A). The results showed that in most cases, DKC1 is a risk factor for various cancers. However, in some specific tumor types, such as ovarian serous cystadenocarcinoma (OV), pheochromocytoma and paraganglioma (PCPG), and stomach adenocarcinoma (STAD), patients with high expression levels had better survival rates. This finding suggested that DKC1 may play different mechanistic roles in different cancers, but this requires further study. To further explain these findings, we used a forest plot to display the Cox survival analysis results for four survival periods (Fig. 8B–E) and used Kaplan‒Meier curves to present the log-rank test results for two cancer types (KIRP and LIHC) and four survival periods (Fig. 8F).

### The correlation of DKC1 with chemotherapy efficacy

**Fig. 9:**
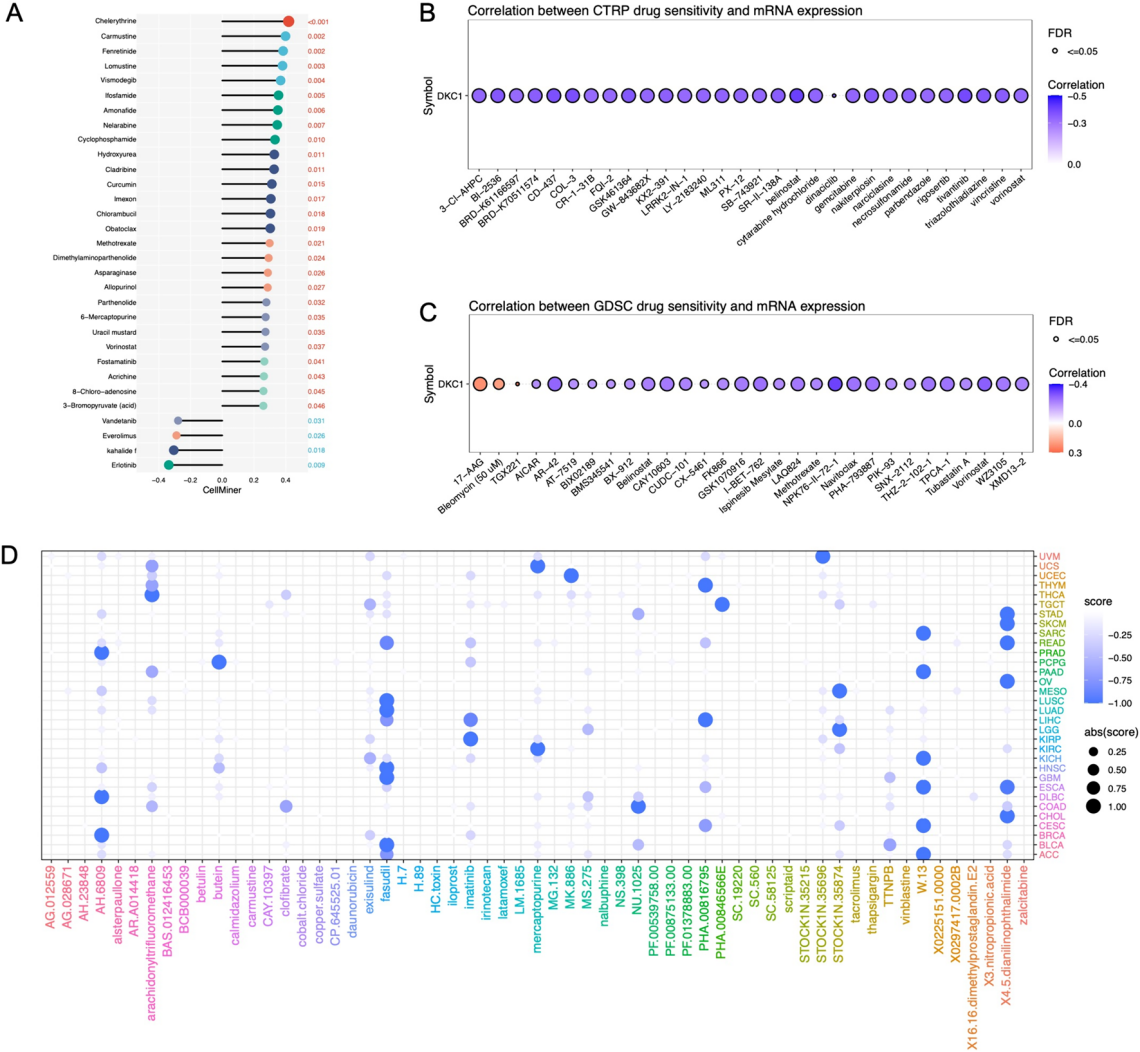
Drug resistance analysis. Drug sensitivity analysis based on DKC1 expression using three different databases: CellMiner (**A**), CTRP (**B**), and GDSC (**C**). A value of *P* < 0.05 indicated statistical significance in the analysis. **D** Prediction of potential compounds targeting DKC1. The candidate compounds were visualized based on connectivity map analysis of 32 cancer types that could target DKC1

In the analysis of chemotherapy efficacy, we investigated the potential association between DKC1 expression and drug sensitivity using three different databases (CTRP, GDSC, and CellMiner). According to the CellMiner database, a positive correlation was observed between DKC1 expression and the sensitivity z scores to numerous drugs (Fig. 9A). In the CTRP and GDSC databases, a significant negative correlation was found between DKC1 and the IC50 values of multiple drugs (Fig. 9B, C). Clearly, DKC1 is potentially associated with chemosensitivity. Furthermore, utilizing the CTD database (Supplementary Table 4), we successfully identified chemical agents capable of modulating DKC1 expression. Among these agents, 62 upregulated DKC1 expression, while 49 downregulated DKC1 expression. To explore potential therapeutic strategies that can counteract the tumor-promoting effects mediated by DKC1, we conducted CMap analysis. We constructed a DKC1-related gene signature, which included the 150 genes whose expression was most significantly upregulated and the 150 genes whose expression was most significantly downregulated, by comparing patients with high DKC1 expression and patients with low DKC1 expression in each cancer type. Using the optimal feature matching method, XSum (eXtreme Sum), we compared the DKC1-related features with the CMap gene features, resulting in similarity scores for 1288 compounds. AH.6809, fasudil, W.13, and X4.5dianilimophthalimide exhibited notably lower scores in most cancer types, suggesting their potential to inhibit DKC1-mediated oncogenic effects (Fig. 9D).

To explore the effect of DKC1 expression on drug sensitivity of ESCA. After treating ESCA cells with different concentrations of Fasudil for 48 h, the CCK-8 method was used to determine that the IC50 of Fasudil on the EC9706 control group was 446.6 μM, and the IC50 in the DKC1 knockdown group of EC9706 was 767.8 μM. In addition, in The IC50 of Fasudil against KYSE30 in the control group was 68.74 μM, and the IC50 in the DKC1 knockdown group of KYSE30 was 79.95 μM (Supplementary Fig. 6). The results showed that after knocking down DKC1, the IC50 for Fasudil in ESCA cells increased.

### Experimental verification in ESCA in vivo and in vitro

**Fig. 10:**
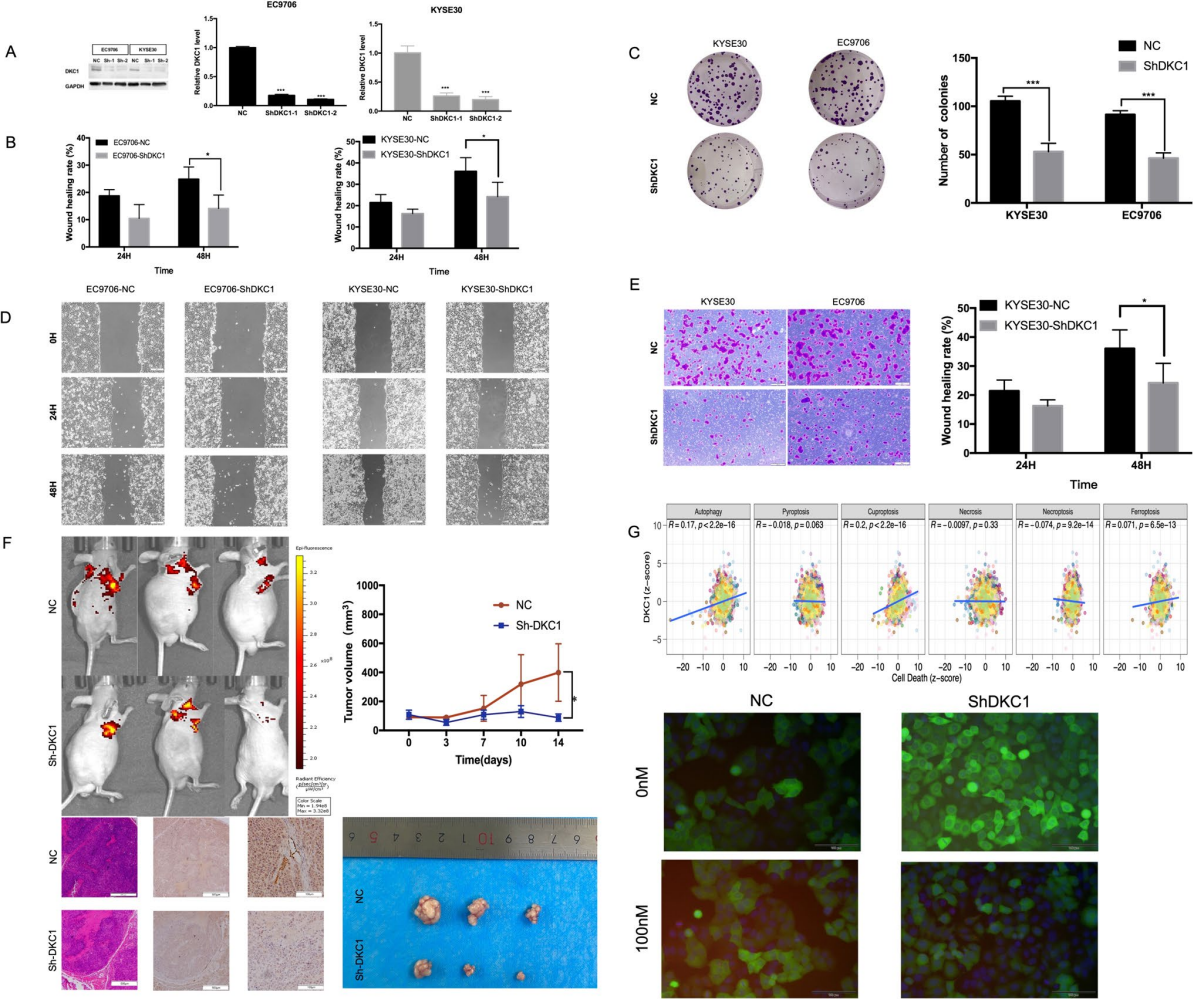
Experimental verification in ESCA in vivo and in vitro. **A** Western blot and qPCR were used to detect the expression of DKC1 protein and mRNA after knocking down DKC1. **B** Wound healing rates were compared between the negative control group and the ShDKC1 group. **C** Cell proliferation was measured by a colony formation assay. **D** Cell migration was measured by a wound healing assay. **E** Cell migration was measured by a transwell assay. **F** Noninvasive imaging of ESCA in live mice, tumor volumes of mice, photographs of tumors, and IHC and HE staining of tumor tissues, *P < 0.05, ***P < 0.001. **G** The correlation between DKC1 expression and cell death and between DKC1 expression and immunofluorescence staining following 100 nM Elesclomol-Cu (1:1 ratio)-induced cuproptosis. (green: GFP; blue: DAPI; red: DLAT)

In order to explore the role of DKC1 in ESCA, we used NC, ShDKC1-1 and ShDKC1-2 lentivirus to infect the cells in ESCA cell lines EC9706 and KYSE30, and then performed puromycin screening. Western blot experiments showed that compared with NC cells, the protein expression of DKC1 in the knockdown group was significantly reduced. And qPCR results showed that DKC1 mRNA levels were significantly reduced in DKC1 knockdown cells compared with NC cells (Fig. 10A). The above results prove that this study successfully constructed cell lines with stable knockdown of DKC1. Subsequently, the colony formation assay (Fig. 10C) revealed that the number of colonies in the ShDKC1 group was significantly lower than that in the control group. We also performed a transwell migration assay (Fig. 9E), which showed that the number of migrating cells in the ShDKC1 group was significantly lower than that in the control group. Furthermore, a wound healing assay was conducted to further validate the effect of DKC1 on migration, and the results showed that the wound healing distance percentage was significantly lower in the ShDKC1 group than in the control group (Fig. 10B, D). These results collectively suggest that DKC1 promotes the proliferation and migration of ESCA cells.

To examine the impact of DKC1 on the proliferative capacity of ESCA in vivo, we established a nude mouse subcutaneous transplantation tumor model by injecting the stable human cell lines EC9706-shNC and EC9706-ShDKC1. Tumor growth was monitored, and the nude mice were sacrificed to excise the tumors for volume measurement. Our findings demonstrated that DKC1 knockdown significantly inhibited tumor growth in nude mice (Fig. 10F).

This study conducted a correlation analysis between various forms of cell death and DKC1 and revealed a positive correlation between DKC1 expression and cuproptosis. Therefore, we induced cuproptosis using Elesclomol-Cu (1:1 ratio) and performed immunofluorescence experiments, which revealed that knockdown of DKC1 resulted in reduced DLAT expression (Fig. 10G), indicating a decrease in cuproptosis.

## Discussion

ESCA poses a formidable threat to public health, ranking as the sixth leading contributor to cancer-related mortality worldwide and the seventh most prevalent disease (Sung et al. 2021). Although there have been some recent advances in treatments, ESCA treatments still need to be improved, as the five-year overall survival rate for patients is less than 40% (Allemani et al. 2018). Therefore, the urgent discovery of new biomarkers for individuals with ESCA and enhanced treatment strategies are imperative (Ju et al. 2022; Wen et al. 2022).

Telomerase exerts a significant influence on cancer progression across the entire spectrum of human life (Maciejowski and de Lange 2017). Telomerase reactivation is observed in most human tumors (Gunes and Rudolph 2013). DKC1 is a component of the telomerase ribonucleic acid protein that maintains telomerase stability and telomere length. Recent studies have shown that dysregulation of DKC1 expression in various human cancers alters cancer cell growth and metastasis (Hou et al. 2020; Miao et al. 2019). Our experimental study revealed the same phenomenon in ESCA.

Recent research has shown that the DKC1 gene is highly expressed in various tumors and influences tumor proliferation and metastasis (Kan et al.^2021^; Liu et al.^2021^; Shang et al.^2023^; Wang et al.^2023^). However, the current understanding of DKC1 is relatively limited, and a comprehensive analysis of its role in cancer has not been conducted. Therefore, this study employed multiple approaches, including differential expression analysis, SCNA, mutation and DNA methylation analysis, immune infiltration analysis, GSEA, survival and clinical outcome analysis, and interaction analysis with chemical substances, to comprehensively analyze the role of DKC1. The findings of this study indicate that high DKC1 expression promotes the progression of various tumors and impacts drug sensitivity. Tumor immunotherapy has made significant progress in the field of cancer treatment (Gross et al.^2023^; Kroemer et al.^2023^). We also discovered a significant correlation between the expression of DKC1 and the expression of immune modulatory molecules, as well as lymphocyte subset infiltration biomarkers. We confirmed that DKC1 is highly expressed in esophageal cancer tissues and cells through in vivo and in vitro experiments and that DKC1 expression is associated with poor patient prognosis. We also found that DKC1 promotes the proliferation and metastasis of esophageal cancer cells. Cuprotosis is a new mode of cell death that relies on copper ion carriers, such as elesclomol, to transport Cu into cancer cells, thereby inducing cell death (Tsvetkov et al. 2022). We demonstrated that DKC1 plays a role in the pathway of cuprotosis. This study suggested that DKC1 may be a potential therapeutic target for cancer therapy.

## Supplementary Information

Below is the link to the electronic supplementary material.

Supplementary file1 (ZIP 3490 KB)

## Data Availability

The TCGA pancancer cohort was obtained from the Firehose database (http://gdac.broadinstitute.org) and accessed through the UCSC Xena browser (https://xenabrowser.net/datapages/).
