# Supplementary figures and images for: Identifying and validating the roles of the cuproptosis-related gene DKC1 in cancer with a focus on esophageal carcinoma
Source: J Cancer Res Clin Oncol. 2024 Aug 5;150(8):382. doi: 10.1007/s00432-024-05870-8 (PMC11300667; doi:10.1007/s00432-024-05870-8)

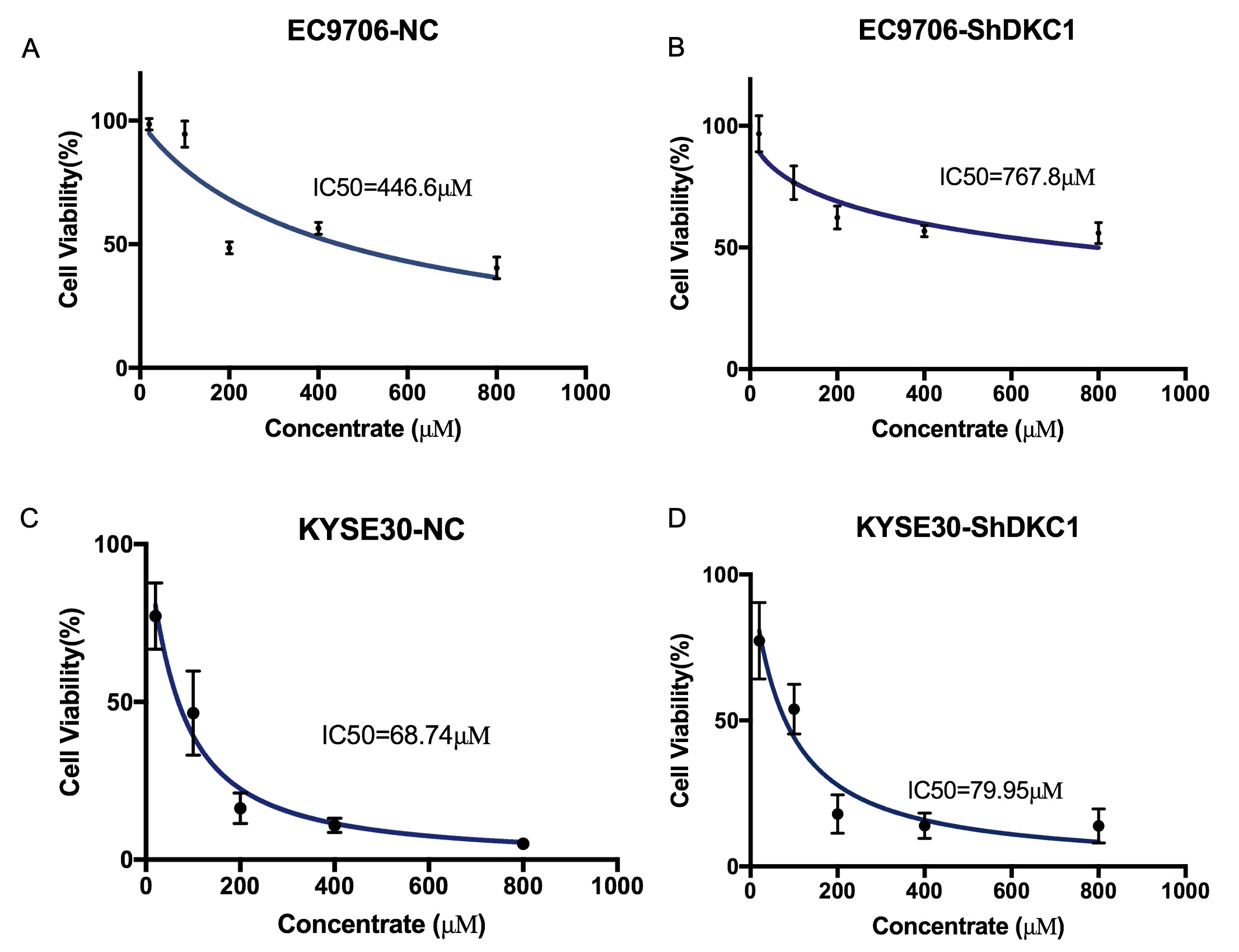

Supplement: Supplementary file 1 — Supplementary file1 (ZIP 3490 KB) [file 432_2024_5870_MOESM1_ESM.zip › Supplementary material/Supplementary Figure 6.jpg]

DKC1 (mRNA Level in GEO)

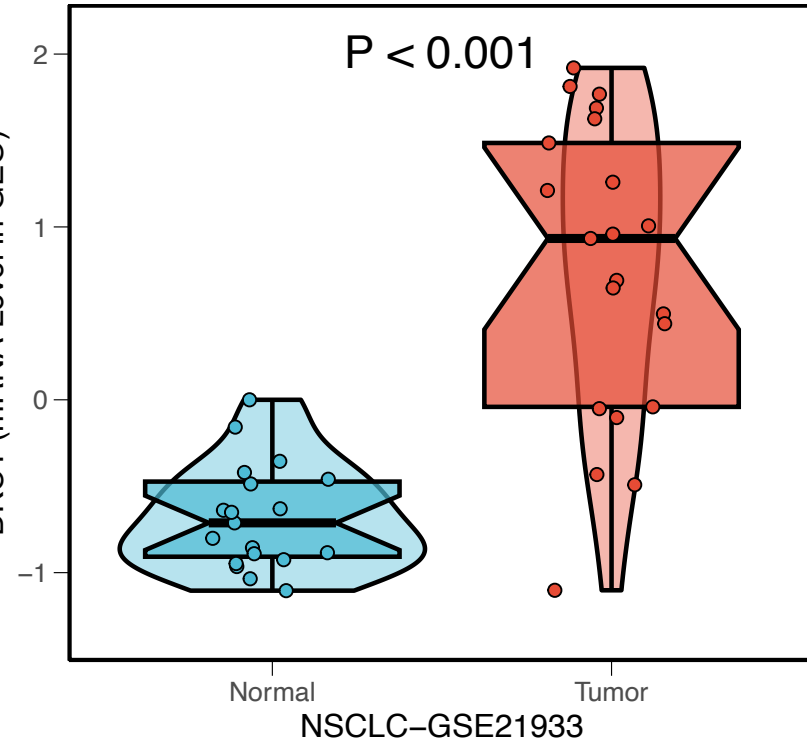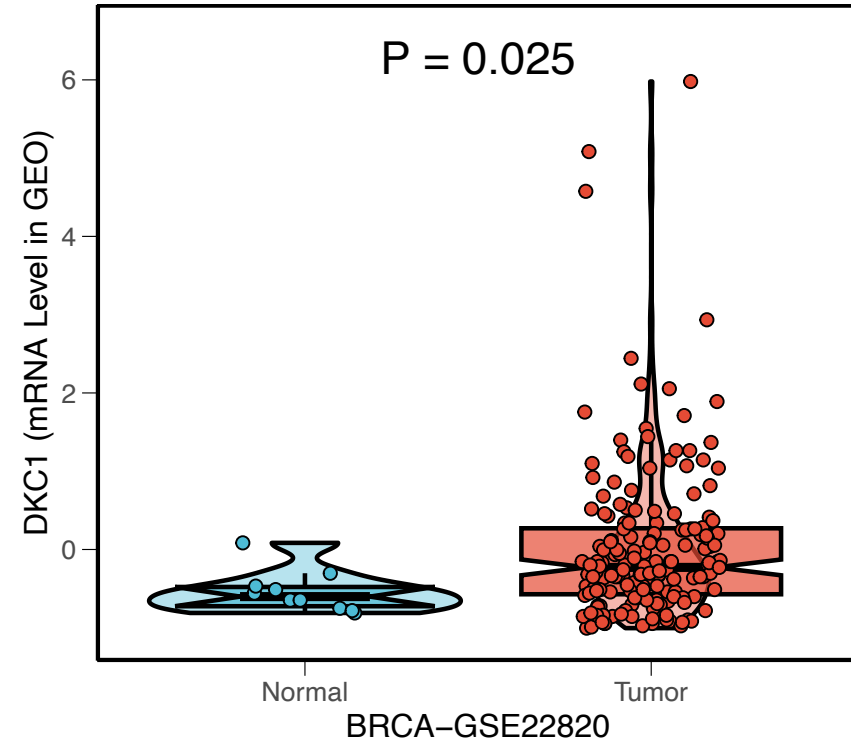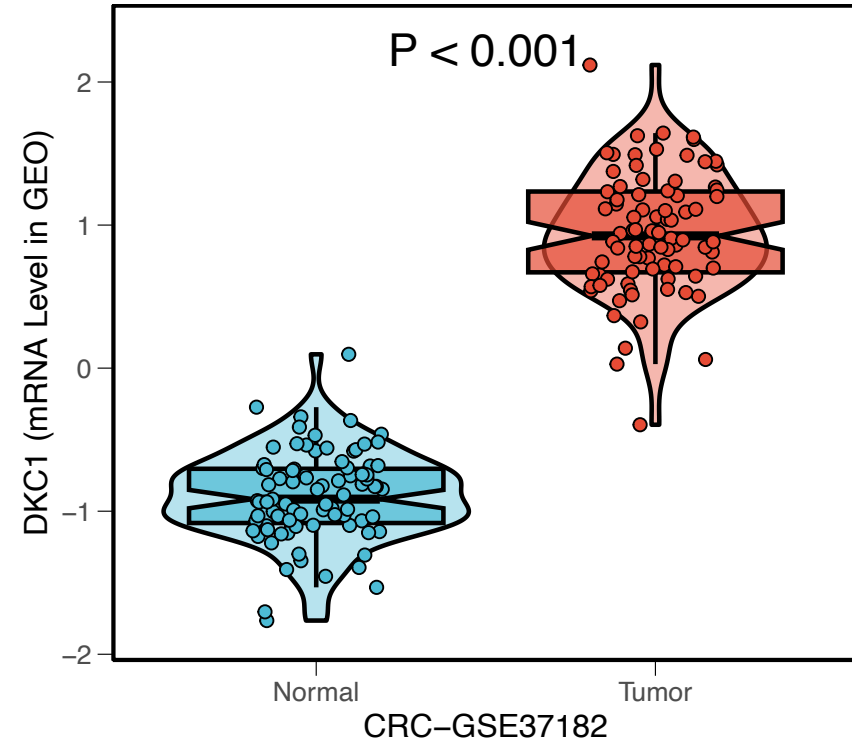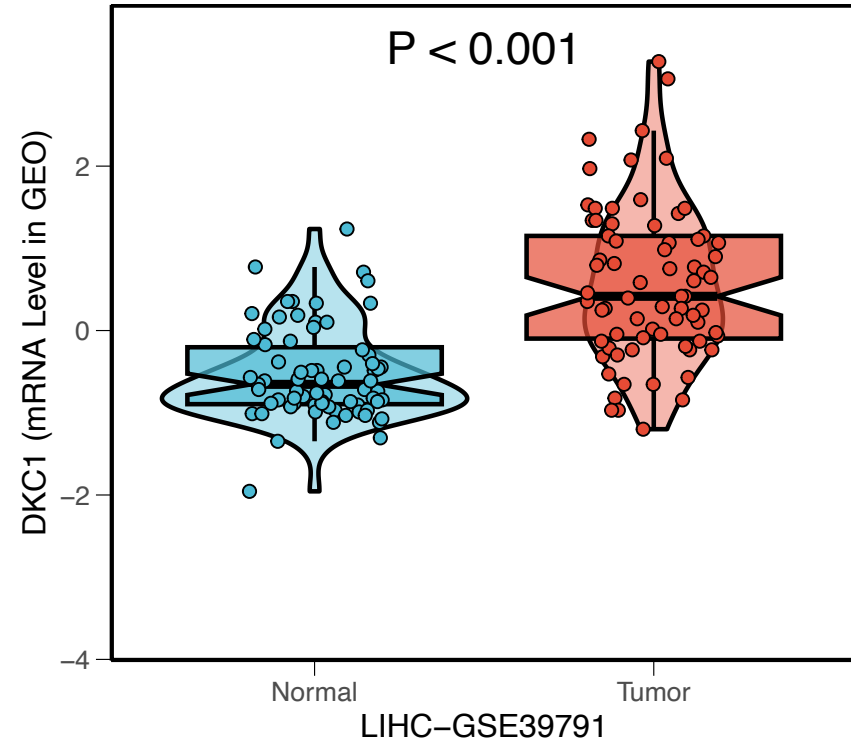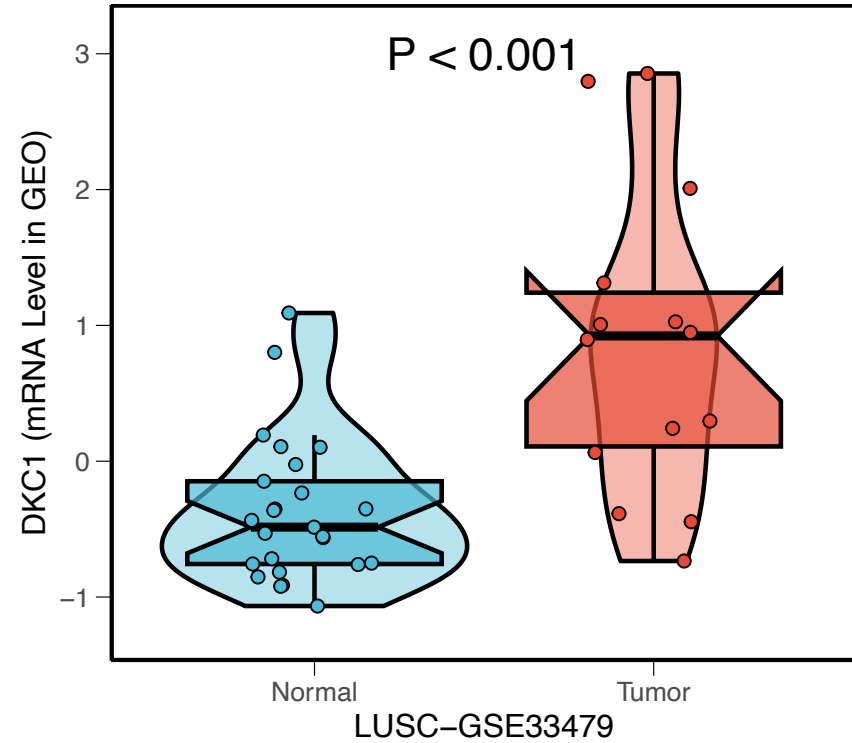

Supplement: Supplementary file 1 — Supplementary file1 (ZIP 3490 KB) [file 432_2024_5870_MOESM1_ESM.zip › Supplementary material/Supplementary Figure3.pdf]

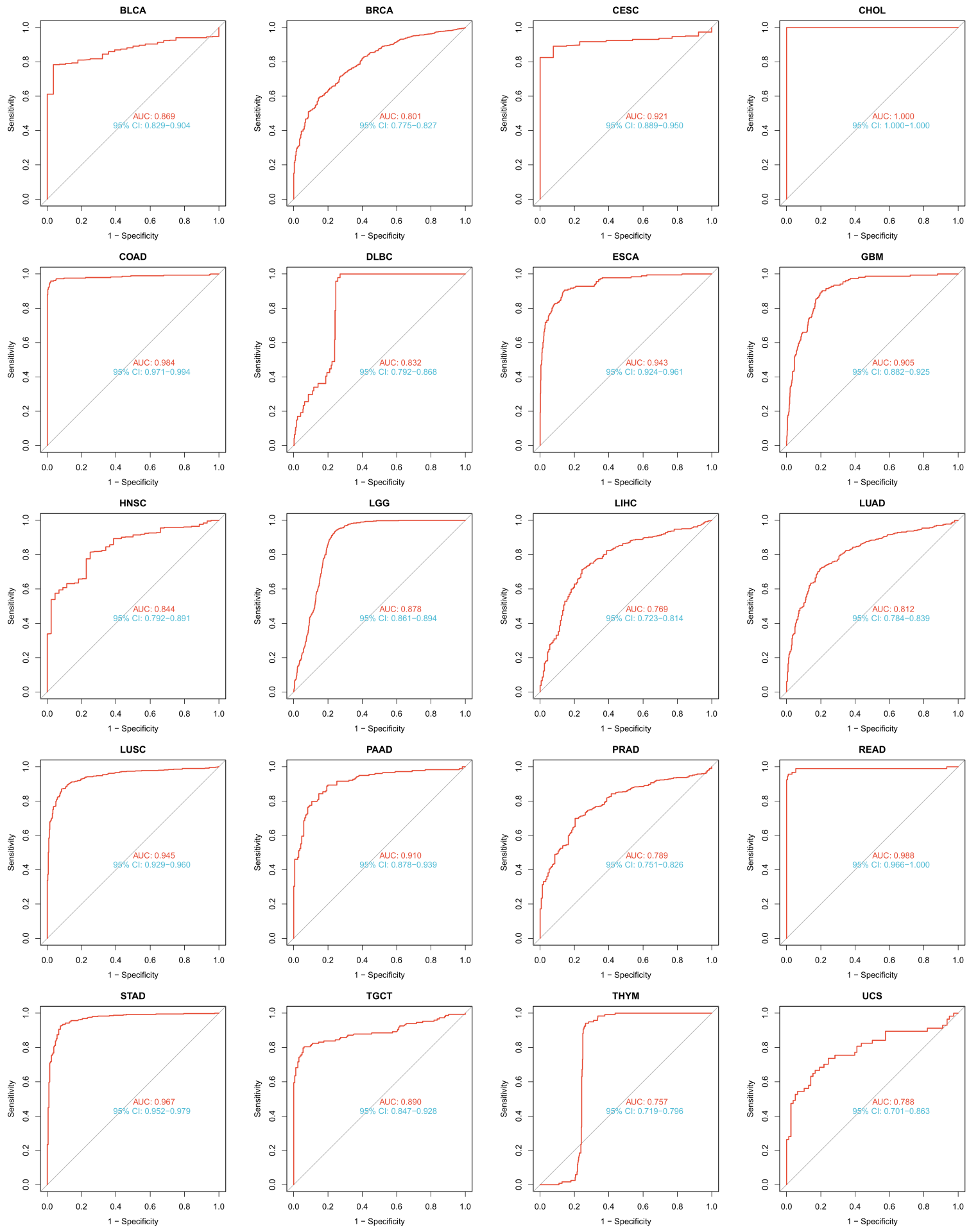

Supplement: Supplementary file 1 — Supplementary file1 (ZIP 3490 KB) [file 432_2024_5870_MOESM1_ESM.zip › Supplementary material/Supplementary Figure 2.pdf]

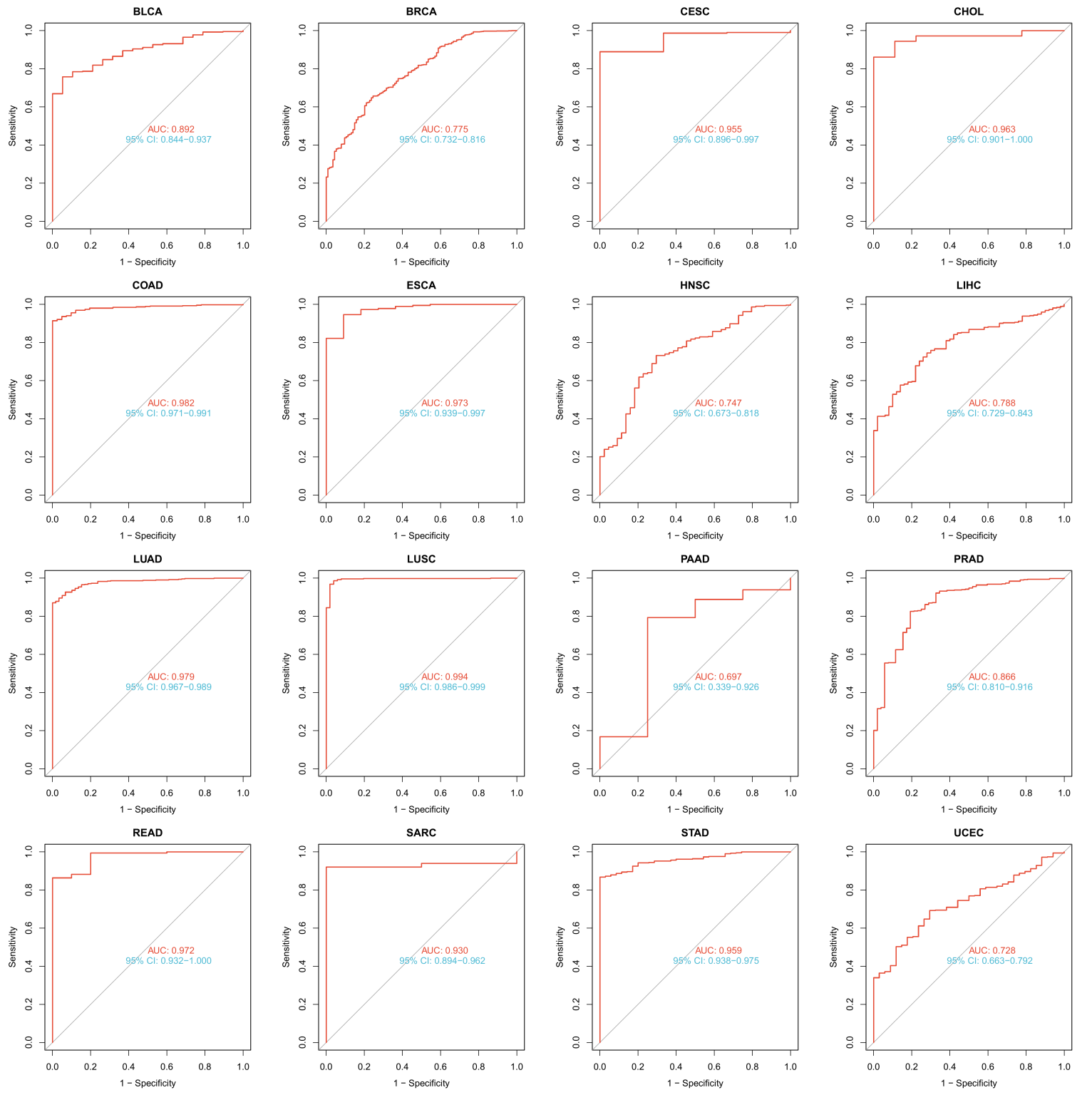

Supplement: Supplementary file 1 — Supplementary file1 (ZIP 3490 KB) [file 432_2024_5870_MOESM1_ESM.zip › Supplementary material/Supplementary Figure 1.ROC.TCGA.pdf]

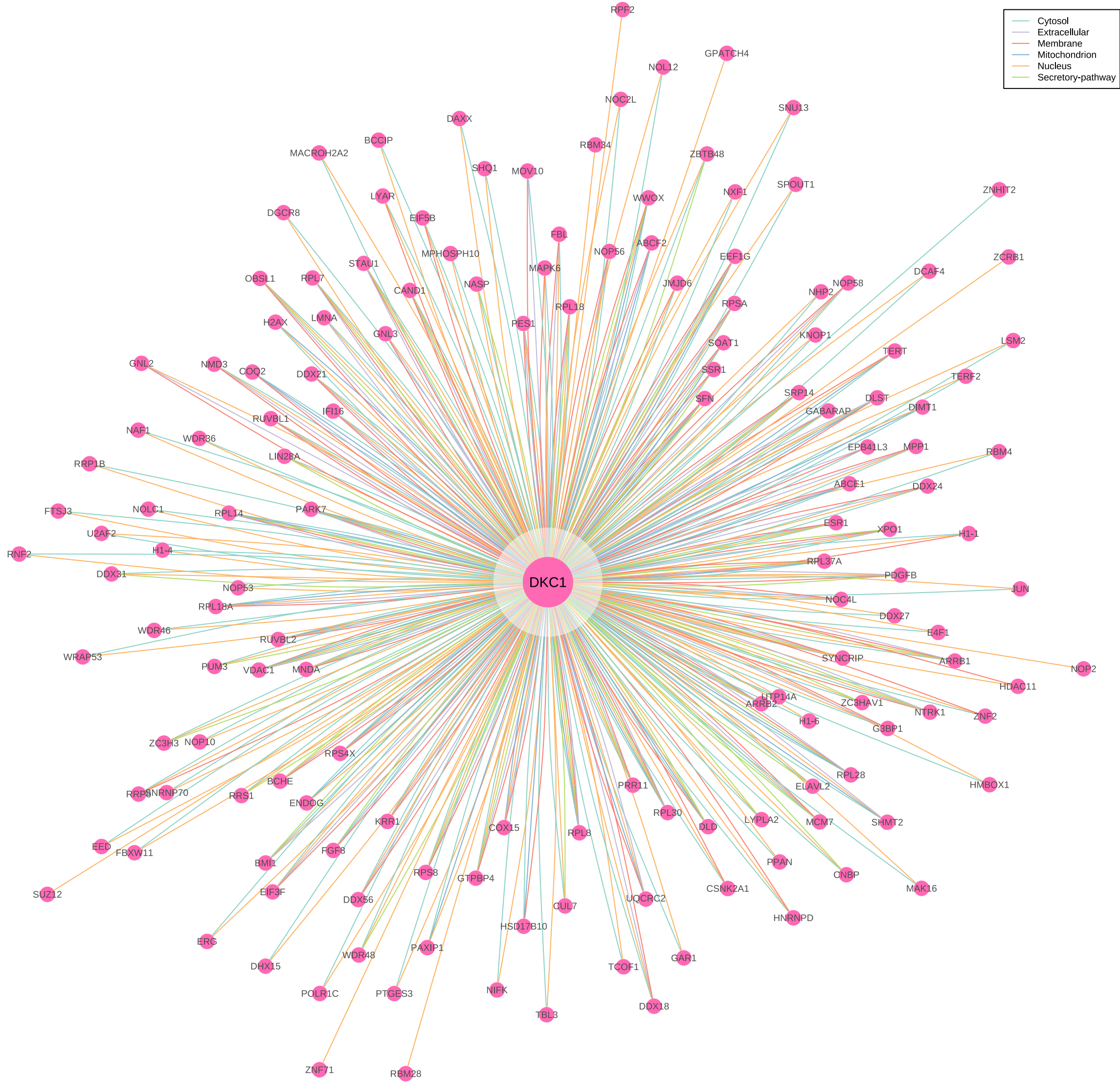

Supplement: Supplementary file 1 — Supplementary file1 (ZIP 3490 KB) [file 432_2024_5870_MOESM1_ESM.zip › Supplementary material/Supplementary Figure4.pdf]

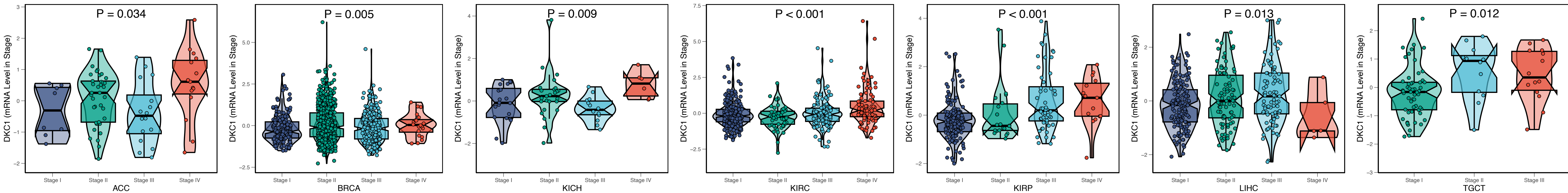

Supplement: Supplementary file 1 — Supplementary file1 (ZIP 3490 KB) [file 432_2024_5870_MOESM1_ESM.zip › Supplementary material/Supplementary Figure5.pdf]
